# Acute Cutaneous Wounds Treated with Human Decellularised Dermis Show Enhanced Angiogenesis during Healing

**DOI:** 10.1371/journal.pone.0113209

**Published:** 2015-01-20

**Authors:** Nicholas S. Greaves, Julie Morris, Brian Benatar, Teresa Alonso-Rasgado, Mohamed Baguneid, Ardeshir Bayat

**Affiliations:** 1 Plastic and Reconstructive Surgery Research, Manchester Institute of Biotechnology (MIB), The University of Manchester, Manchester, Lancashire, United Kingdom; 2 University Hospital of South Manchester NHS Foundation Trust, Wythenshawe Hospital, Manchester, Lancashire, United Kingdom; 3 Department of Histopatholgy, Pennine Acute Hospitals NHS Trust, Royal Oldham Hospital, Rochdale Road, Oldham, Lancashire, United Kingdom; 4 School of Materials, University of Manchester, Manchester, Lancashire, United Kingdom; 5 Centre for Dermatology, Institute of Inflammation and Repair, Faculty of Medicine and Human Sciences, University of Manchester, Manchester Academic Health Science Centre, Manchester, United Kingdom; University of New Mexico HSC, UNITED STATES OF AMERICA

## Abstract

**Background:**

The influence of skin substitutes upon angiogenesis during wound healing is unclear.

**Objectives:**

To compare the angiogenic response in acute cutaneous human wounds treated with autogenic, allogenic and xenogenic skin substitutes to those left to heal by secondary intention.

**Methods:**

On day 0, four 5mm full-thickness punch biopsies were harvested from fifty healthy volunteers (sites 1-4). In all cases, site 1 healed by secondary intention (control), site 2 was treated with collagen-GAG scaffold (CG), cadaveric decellularised dermis (DCD) was applied to site 3, whilst excised tissue was re-inserted into site 4 (autograft). Depending on study group allocation, healing tissue from sites 1-4 was excised on day 7, 14, 21 or 28. All specimens were bisected, with half used in histological and immunohistochemical evaluation whilst extracted RNA from the remainder enabled whole genome microarrays and qRT-PCR of highlighted angiogenesis-related genes. All wounds were serially imaged over 6 weeks using laser-doppler imaging and spectrophotometric intracutaneous analysis.

**Results:**

Inherent structural differences between skin substitutes influenced the distribution and organisation of capillary networks within regenerating dermis. Haemoglobin flux (p = 0.0035), oxyhaemoglobin concentration (p = 0.0005), and vessel number derived from CD31-based immunohistochemistry (p = 0.046) were significantly greater in DCD wounds at later time points. This correlated with time-matched increases in mRNA expression of membrane-type 6 matrix metalloproteinase (MT6-MMP) (p = 0.021) and prokineticin 2 (PROK2) (p = 0.004).

**Conclusion:**

Corroborating evidence from invasive and non-invasive modalities demonstrated that treatment with DCD resulted in increased angiogenesis after wounding. Significantly elevated mRNA expression of pro-angiogenic PROK2 and extracellular matrix protease MT6-MMP seen only in the DCD group may contribute to observed responses.

## Introduction

Angiogenesis is a crucial mechanism during wound healing involving the dynamic co-ordinated interaction of structural, cellular and molecular components [1]. Defined as formation of new capillaries from pre-existing blood vessels, this key component of the proliferative phase gives rise to vasculature forming up to 60% of granulation tissue [2, 3]. Normally, angiogenic stimulation results in nitric oxide dependent vasodilatation and increased vascular permeability in response to vascular endothelial growth factor (VEGF) [4]. Subsequent extravasation of plasma proteins forms a provisional scaffold for endothelial cell migration, facilitated by secretion of matrix metalloproteinases and angiopoietin-2 which degrade the extracellular matrix (ECM) and liberate further growth factors [3, 4]. Endothelial cells behind the migratory front proliferate, elongating capillary sprouts forming cord-like structures. Newly formed vessels are stabilised by recruitment of smooth muscle cells, pericytes, fibroblasts and secretion of ECM proteins whilst lumen formation is dependent upon VEGF, angiopoietin-1 and integrins [3, 4].

After injury, an abundant blood supply is required to fuel the increased local metabolic demands of the healing process, whilst endothelial cells themselves are pivotal co-ordinators of fibroplasia and ECM remodelling [2]. Unbalanced regulation of angiogenesis can result in abnormal scarring, delayed wound healing and chronic wound formation. Indeed, down-regulated or ineffectual angiogenic drive is a recognised pathogenic mechanism in venous and diabetic ulcers whilst stimulation of angiogenesis has been shown to enhance healing rates in diabetic subjects [5–8].

Treatment options within the chronic wound field have expanded significantly with the introduction of dermal skin substitutes (dSS). These bioengineered materials have variable design depending on their source (autograft, allograft or xenograft) and cellular content (acellular versus cellular) [9]. Fundamentally, they act as biocompatible ECM equivalents that integrate into the wound bed to stimulate revascularisation, cellular migration and repopulation of injured tissue [9]. The evidence for using dSS in chronic wound management is increasing with randomised controlled trials and a Cochrane review demonstrating improved healing rates in diabetic and venous ulcers compared to existing treatment regimes [10–14]. However, these evaluation studies did not elucidate mechanisms for observed improvements. We recently showed application of human decellularised dermis (DCD) to treatment-resistant leg ulcers resulted in complete healing in 60% of cases. Increased wound bed haemoglobin flux and CD31 values suggested DCD-related up-regulation of angiogenesis contributed to successes observed [15]. Investigation of blood vessel development within dSS and the influence of such materials on angiogenesis after wounding is still poorly understood and restricted to animal models [16, 17]. Furthermore, it is not clear whether pro-angiogenic effects of dSS are limited to cases where healing is impaired such as chronic wounds or whether the same influence is observed in acute wounds.

In this unique human clinical study, we quantitatively compared the angiogenic response to wounding in 50 healthy volunteers, treated with different dSS (DCD, collagen-GAG scaffold (CG) and autograft) and compared them to secondary intention wound healing (Control). Using correlating invasive and non-invasive assessment tools, we successfully investigated whether the angiogenic influence of dSS seen in chronic wounds was translated to the acute wound setting, established whether this behaviour was dependent upon dSS characteristics and provided supporting experimental data for observed behaviour.

## Materials and Methods

### Study methodology

National Research Ethics Service (NRES reference number 12/NW/0078) and local research and development department (University Hospital South Manchester NHS Foundation Trust, reference number 2011BP001) approval was granted. Fifty healthy volunteers meeting study inclusion/exclusion criteria (Table 1) gave informed written consent to participate. Recruits included 26 females and 24 males (mean age 26 years) who were electronically randomised into one of five study groups, each containing 10 patients.

**Table 1:** Study inclusion and exclusion criteria.

**Inclusion Criteria**	**Exclusion Criteria**
Aged 16 or over	Subjects of either gender who are under 16 years old
Able to understand study requirements and attend all follow-up visits	Any subject, who in the opinion of the investigator is unable to fully understand the requirements of the trial, consent or is unable to return to follow-up visits in order to complete the study
Able to provide written consent if competent	Subjects who do not give consent or withdraw their consent to take part in the study
Weight between 40–150kg with a body mass index of 20–35kg/m^2^	Subjects who have a history of keloid or hypertrophic scarring
	Subjects who take medication known to influence/alter the healing of skin (e.g steroids)
	Subjects who are receiving formal oral anticoagulant therapy (e.g warfarin)
	Subjects who have taken part in clinical studies or received any investigational drugs 2 months prior to day 0
	Subjects who have evidence of drug abuse
	Subjects who have had or are known to have hepatitis B or hepatitis C infection including carriers of hepatitis B surface antigen, hepatitis B core antibodies or hepatitis C antibodies. Previous vaccination against hepatitis B or C is not excluded
	Subjects who have previously had a positive result to the HIV antibody test or admit to belonging to a high risk group.
	Subjects who become systemically unwell during the research process due to external study causes.
	Subjects with known allergies to antibiotics
	Pregnant subjects or those likely to become pregnant in the next 3 months
	Subjects with active skin disorders considered to adversely affect wound healing by the investigators (e.g dermatitis, psoriasis etc)

**Figure 1:**
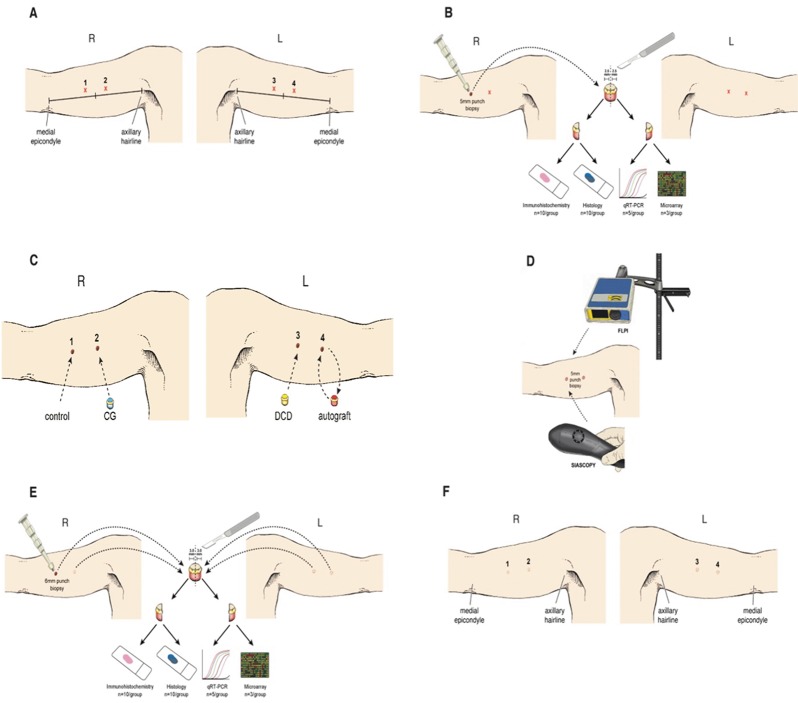
Cohort study methodology. On day 0, 2 sites on each upper arm were allocated (A). Sites were scanned using full-field laser perfusion imaging (FLPI) and spectrophotometric intracutaneous analysis (SIAscopy), before 5mm punch biopsies were taken under local anaesthetic (B). Excised tissue was processed using a range of laboratory techniques. Site 1 was left to heal by secondary intention, site 2 was filled with a 5mm disc of collagen-GAG scaffold (CG), site 3 treated with a 5mm disc of human decellularised dermis (DCD) while excised tissue from site 4 was replaced in the defect acting as an autograft (C). Subjects were seen weekly for 6 weeks where the wound was assessed clinically and non-invasive scanning repeated (D). Subjects subsequently underwent 6mm excision punch biopsies of healing tissue based on study group allocation (E). Excised tissue was again processed using a range of laboratory techniques. All wounds in the study healed satisfactorily leaving a small scar (F).

On day 0, anatomical sites on each arm (sites 1–4), located 2cm proximal and 2cm distal to the mid-point of a line connecting the axillary hairline with the medial epicondyle were assigned (Fig. 1A). Skin at these locations was investigated using full-field laser perfusion imaging (FLPI—Moor Instruments, Axminster, UK) and spectrophotometric intracutaneous analysis (SIAscopy—Phycon Medical Inc, Miami, USA) providing baseline data for normal skin. FLPI enables non-invasive measurement of blood flow (haemoglobin flux) in skin microcirculation and indirect assessment of angiogenesis after treatment using a monochromatic laser. Tissue thickness sampled is typically 1mm and capillary diameters of up to 10μm with flow rates of 0.01–10mm/s can be detected. SIAscopy provides in-vivo measurement of oxyhaemaglobin through cutaneous spectral probing using light ranging from 400–700nm wavelength [15].

Five millimetre full-thickness punch biopsies were taken from allocated sites under local anaesthetic (Bupivacaine 0.5% AstraZeneca, Macclesfield, UK) (Fig. 1B). Full thickness was defined as removing the entire epidermis and dermis to expose hypodermic fat. After haemostasis, site 1 healed by secondary intention (Control), site 2 was filled by a 5mm disc of single layered collagen-glycosaminoglycan wound matrix scaffold (termed “CG”) (Integra Life Sciences, Plainsboro, NJ, USA), site 3 was filled with a 5mm disc of National Health Service Blood and Transplant derived Decellularised Dermis (DCD) (NHSBT, Watford, UK) and tissue excised from site 4 was left ex-vivo for two minutes before being replaced intact back into the defect (Autograft) (Fig. 1C). Once hydrated in sterile saline, CG and DCD were of similar thickness prior to implantation (<1mm) whilst autografts were inherently thicker (>1mm) as they were derived from full-thickess biopsies. Wounds were covered with Tegaderm (3M, Bracknell, UK) for 7 days and patients given standard wound care advice.

**Figure 2:**
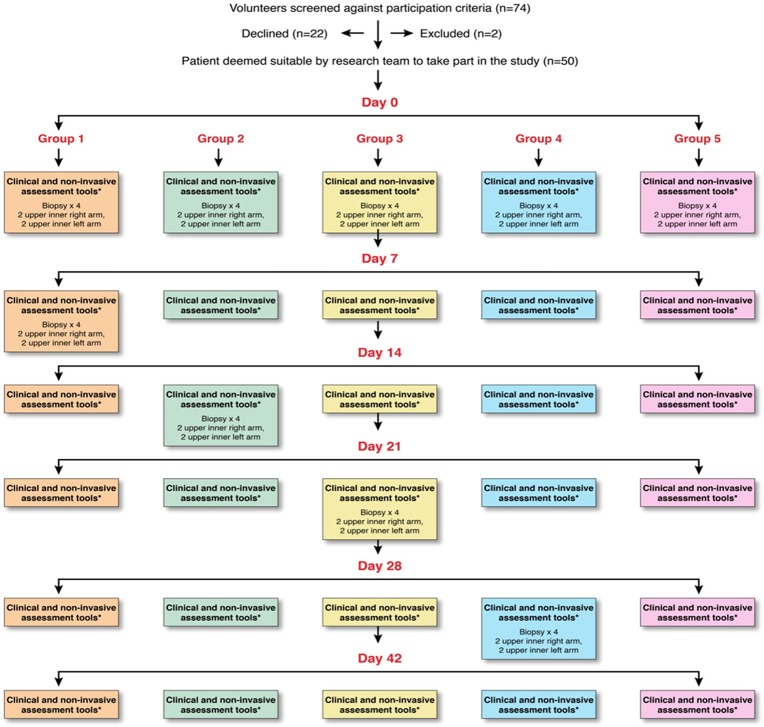
Cohort study flowchart. 74 volunteers were screened. 2 subjects did not fulfil recruitment criteria and were excluded. A further 22 declined to take part in the study. 50 participants were enrolled and randomly allocated to one of 5 groups each containing 10 patients. All groups had 4 punch biopsies harvested on day 0 before undergoing dermal skin substitute or control treatment. Group number determined the time point for excision biopsies of healing tissue. Group 1 at day 7, group 2 at day 14, group 3 at day 21, group 4 at day 28 and group 5 did not have repeat biopsies. Volunteers were reviewed weekly for 6 weeks post day 0 and wounds assessed serially using non-invasive measures. Complete data for each investigative modality at each time point was obtained with no loss to follow-up.

Patients attended follow-up on day 7, 14, 21, 28 and 42 where wounds were assessed using non-invasive tools (Fig. 1D). FLPI and SIAscopic measurements were taken in triplicate at each wound site with average values used in final analysis. Group 1 patients underwent 6mm full thickness excision biopsies of healing tissue at sites 1–4 on d7, group 2 on d14, group 3 on d21 and group 4 on d28 (Fig. 1E). Group 5 patients did not undergo excision biopsies (Fig. 2). All wounds healed successfully (Fig. 1F).

### Laboratory techniques

**Histology (n = 10 patients/group).** Formalin-fixed paraffin-embedded tissue sections at a thickness of 3µm were prepared on glass slides. Haemotoxylin and eosin staining (H&E) was performed using Tissue-Tek Prisma automated stainer (Sakura Finetek Europe, Alphen aan den Rijn, Netherlands). Slides were dewaxed with xylene (Sigma-Aldrich, Gillingham, UK) and rehydrated before being stained with Harris haematoxylin (CellPath, Newtown, UK) for 7 minutes. Excess haematoxylin was removed by washing in a 0.05% solution of acid alcohol for 10 seconds. Nuclei were then blued using Scott’s tap water substitute for 1 minute and 15 seconds. Tissue sections were stained with alcoholic eosin (CellPath) for 2 mins. Following staining, slides were dehydrated in 99% industrial methylated spirit and rinsed in xylene before being mounted using KP coverslipping tape (Klinipath, Olen, Belgium).

**Immunohistochemical staining (n = 10 patients/group).** Formalin-fixed, paraffin-embedded tissue sections at a thickness of 3µm were mounted onto positively-charged glass slides (Menzel-Glaser, Berlin, Germany). All immunohistochemical steps were performed using Ventana Benckmark Ultra automated stainers (Ventana Medical Systems, Tucson, AZ, USA) and UltraView Universal DAB Detection Kits (Ventana Medical Systems). Washing steps were performed between each step of the protocol using Reaction Buffer (Ventana Medical Systems). Tissue sections were dewaxed by heating slides to 60°C and incubating in EZ Prep solution (Ventana Medical Systems) for 4 minutes followed by 3 rinses with EZ Prep solution. Antigen retrieval was performed as per the protocol in table 2. Endogenous peroxidase was blocked by incubating slides in UV Inhibitor (Ventana Medical Systems) for 4 minutes at room temperature. Primary antibody was applied to slides and tissue sections were incubated at 36°C for 36 minutes. Slides were then incubated in UV HRP Universal Multimer (Ventana Medical Systems) for 8 minutes at 36°C. One drop of UV DAB and UV DAB H2O2 (Ventana Medical Systems) were then applied to tissue sections and incubated at room temperature for 8 minutes. DAB chromogen was toned by incubating sections in UV Copper (Ventana Medical Systems) for 4 minutes at room temperature. Tissue sections were counterstained by incubating slides in Haematoxylin II (Ventana Medical Systems) for 8 minutes followed by incubation in Bluing Reagent (Ventana Medical Systems) for 4 minutes. Following staining, slides were washed in EZ Prep solution, washed in tap water, dehydrated in 99% industrial methylated spirit and rinsed in xylene (Sigma-Aldrich) before being mounted using KP cover-slipping tape (Klinipath). Definiens Tissue Studio software version 3.5.1 (Definiens, Munich, Germany) was used for CD31 stain analysis.

**Table 2:** Antibody and staining regime used in CD31 immunohistochemical staining.

**Antibody**	**Antibody clone**	**Producer**	**Antibody dilution**	**Antigen retrieval reagent**	**Antigen retrieval temperature (°C)**	**Antigen retrieval duration (mins)**	**Antibody incubation (mins)**
CD31	JC70A	Dako	1:50	CC1	95	36	32

**RNA extraction (n = 5 patients/group).** Tissue from day 0 and extraction biopsies were homogenised in 300μL of RLT buffer (Qiagen, GmbH, Helden, Germany) mixed with β-mercaptoethanol (Sigma-Aldrich, St Louis, MO) (10μl β-mercaptoethanol/ml RLT buffer) using 5-mm stainless steel beads and a Qiagen TissueLyser II (Qiagen GmbH) at 30 oscillations/second for 10 minutes. RNA was extracted using RNeasy Fibrous Tissue Mini Kits (Qiagen GmbH) and Qiagen Qiacube (Qiagen GmbH). Eluted RNA quantity and purity was assessed using NanoDrop ND-100 v3.0.1 spectrophotometer (NanoDrop Technologies, Wilmington, DE) and RNA integrity measured via Agilent 2100 Bioanalyser (Agilent Technologies, Wilmington, DE).

**Whole genome microarray (n = 3 patients/group).** Agilent Whole Human Genome (8×60K) Oligo Microarrays (Agilent Technologies) containing 50,599 features and the manufacturer’s protocol titled One-Colour Microarray-Based Gene Expression Analysis (Low Input Quick Amp Labelling—Version 6.5 May 2010) were employed. 50ng RNA templates were used to create labelled complimentary-RNA using Quick Amp Labelling Kits (One-Colour) (Agilent Technologies). Gene Expression Hybridisation Kits (Agilent Technologies) were utilised for 17-hour array hybridisation, following which arrays were washed with Gene Expression Wash Buffer Kit stabilisation and drying solution (Agilent Technologies). Arrays were scanned at 5μM scan resolution using Axon GenePix 4400A (Molecular Device Ltd, Wokingham, UK) and images extracted using GenePix Pro 7 (Molecular Device Ltd). Normalised data was analysed using Qlucore Omics Explorer 2.3 (Qlucore, Lund, Sweden). Comparisons between patient-matched d0 biopsies (n = 15) and subsequent time points for each treatment group (n = 3) were performed. For each comparison, the top 500 genes with p and q-values of <0.05 were selected.

**qRT-PCR (n = 5 patients/group).** To validate microarray results, expression of selected candidate genes and a reference gene was studied using qRT-PCR. 1μg RNA was used as template to synthesise complementary DNA (cDNA) using qScript cDNA Synthesis Kit (Quanta Biosciences, Gaithersburg, MD). Ribosomal protein L32 (RPL32) was used as a reference gene for normalisation. PCR reactions were carried out in triplicate using the Roche LightCycler 480 system (Roche Diagnostics GmBh) as explained previously [18]. Negative control reactions included all PCR components, but used water as a template instead of a cDNA aliquot.

### Dermal Skin Substitutes

**National Health Service Blood & Transplant derived Decellularised Dermis (DCD).** DCD is a tissue allograft licensed for human application/transplantation and approved by the UK Human Tissue Authority. DCD is prepared from donated cadaveric split thickness skin grafts. Sequential treatments are applied to remove the epidermis and cellular components of the dermis as described by Hogg et al before it is terminally sterilised [19].

**Collagen-Glycosaminoglycan Wound Matrix Scaffold (CG).** Integra Matrix Wound Dressing is a single layered acellular dermal replacement comprising a porous matrix of cross-linked type I bovine tendon collagen and shark chondroitin-6-sulphate. Integra is licensed for multiple indications including venous and diabetic ulcers and gained US Food and Drug Administration approval in 2002.

### Statistics

Initial analysis was performed that tested for an “arm effect” (left arm versus right arm) in order to rule out site-specific variations. For each variable, testing showed there was no significant difference between the mean response on the left arm and the mean response on the right arm. On the basis that the arm effect was not statistically significant, a second analysis (listed below) was performed for each investigational modality that looked for differences between treatment groups.

**SIAscopy, FLPI and Immunohistochemistry.** Differences between treatments at each time point were compared using ANOVA with Greenhouse-Geisser method. Time points with significant treatment differences underwent additional pair-wise comparisons and Bonferroni adjustments for multiple testing were applied. Conventional two-sided 5% significance level was used.

**qRT-PCR.** Non-parametric Friedman tests compared fold changes between treatments at days 7, 14, 21 and 28 separately. If significant differences were found, paired Wilcoxon rank sum tests were utilised to analyse differences between specific types. Significant changes within treatment groups over time were assessed using one-sample Wilcoxon signed-rank tests. Conventional two-sided 5% significance levels were used.

## Results

### Non-invasive imaging demonstrates significantly increased haemoglobin flux and oxyhaemoglobin concentration in DCD samples compared to controls

**Figure 3:**
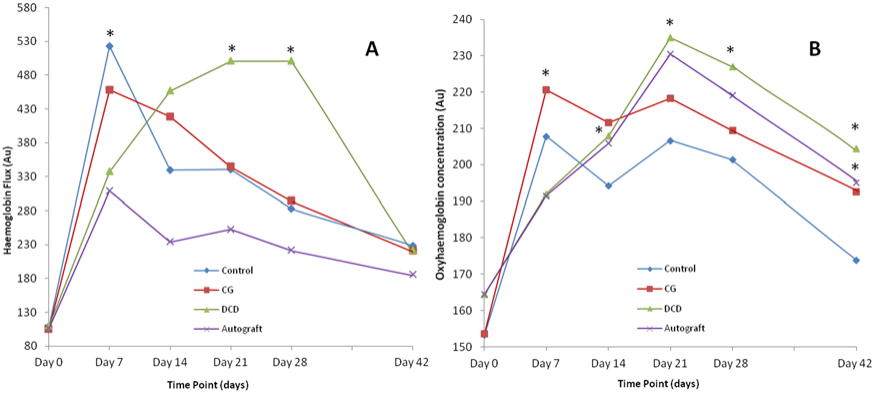
Haemoglobin flux and oxyhaemoglobin concentration. Change in haemoglobin flux (A) and oxyhaemoglobin concentration (B) after wounding in all treatment groups derived from full-field laser perfusion imaging (FLPI) and spectrophotometric intracutaneous analysis (SIAscopy) respectively. Non-invasive imaging confirms significantly up-regulated haemoglobin flux and oxyhaemoglobin concentration after treatment with DCD compared to controls. * p<0.05 n = 10/treatment group.

FLPI and SIAscopy were used to non-invasively assess wound sites before, and at weekly intervals after punch biopsies with associated skin substitute treatments. Seven days (d) post-wounding, mean haemoglobin flux derived from FLPI in DCD and autograft groups was significantly lower than controls (p = 0.0008 and p = 0.0001 respectively). However, this reversed by d21 and d28, with the DCD group showing significantly increased haemoglobin flux compared to controls (p = 0.0035 and p<0.0001 respectively) (Fig. 3A). SIAscopy showed that seven days post-wounding, mean oxyhaemoglobin in CG wounds was significantly greater than in controls (p = 0.0007). However, at all subsequent time points mean oxyhaemoglobin was significantly raised in the DCD group compared to controls (p = 0.0031 (d14), p = 0.0004 (d21), p = 0.0005 (d28), and p<0.0001 (d42) respectively). Likewise, mean haemoglobin was significantly greater in autografts 6 weeks after injury compared to controls (p = 0.0028) (Fig. 3B).

### Structural differences between treatment modalities strongly influence development and organisation of vascular architecture within healing tissue

**Figure 4:**
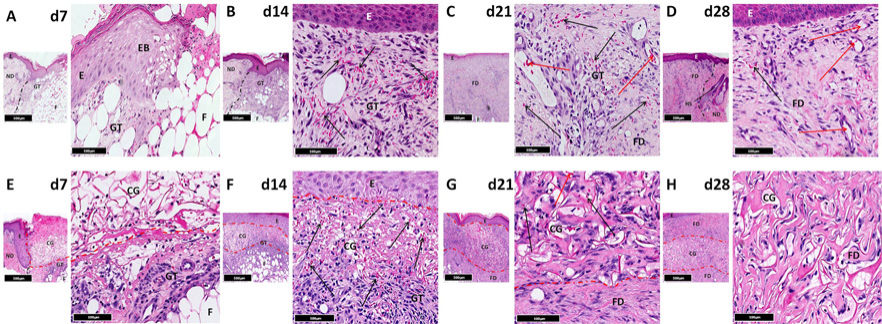
Histological evaluation. Characteristic histological changes in acute cutaneous wounds left to heal by secondary intention (control) (2A-D) and treated with collagen-GAG scaffold (CG) (2E-H) focussing on blood vessel distribution and organisation. In both cases there is rapid development of granulation tissue with subsequent fibrosis, however CG samples develop fewer large vessels than controls. CG—collagen-GAG scaffold, E—epidermis, EB—epidermal bulge, F—subcutaneous fat, FD—fibrotic dermis, GT—granulation tissue, HS—hair shaft, ND—native dermis. Red arrow—patent vessel lumen, Black arrow—granulation tissue vessels. Black dotted line—demarcates border between normal tissue and healing tissue. Red dotted line—demarcates CG. Smaller image—x5 magnification, larger image—x20 magnification.

**Figure 5:**
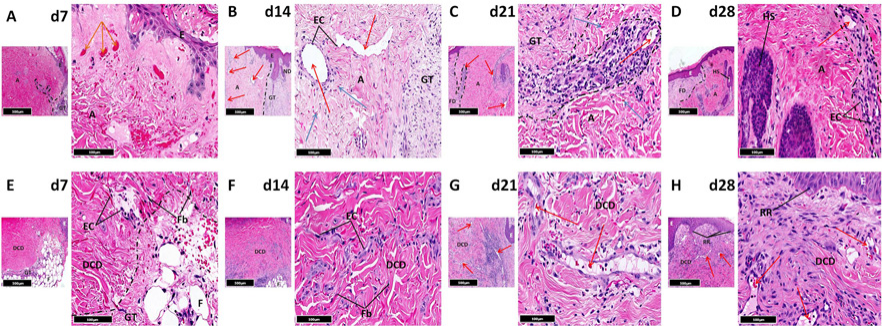
Histological evaluation. Characteristic histological changes in acute cutaneous wounds treated using autografts (2A-D) and DCD (2E-H) focussing on blood vessel distribution and organisation. There is evidence of host migration through defined entry points in both grafted materials promoting revascularisation in autografts and rapid development of capillary networks in DCD. A—autograft, DCD—decellularised dermis, E—epidermis, EC—endothelial cells, Fb—fibroblast, FD—fibrotic dermis, GT—granulation tissue, ND—native dermis, RR—rete ridge. Red arrow—patent vessel lumen, Blue arrow—granulation tissue vessels, Orange arrow—dermal red blood cell extravasation. Black dotted line—border between autograft and host tissue, with finger like projections of inflammatory granulation tissue into autograft or DCD facilitating re-establishment of vascular channels. Smaller image—x5 magnification, larger image—x20 magnification.

Punch biopsies of healing tissue from all 4 wound sites in each patient were excised at time points determined by study group allocation. Harvested tissue was stained with haemotoxylin and eosin facilitating time-matched histological comparison of controls and treatment groups. In control wounds, there was progressive formation of granulation tissue in the wound bed after injury (Fig. 4A). After 14 days, it was densely cellular and had expanded significantly with formation of small vessels and evidence of fibroplasia (Fig. 4B). By d21, deeper granulation tissue had begun to mature and become fibrotic whilst superficial regions still contained numerous vessels. Concurrently, large mature vessels were observed for the first time (Fig. 4C). By d28, dermal fibrosis was established with hyalinisation and associated vascular regression (Fig. 4D). In contrast, CG absorbed extravasated blood and fibrinous exudate after injury resulting in expansion of spaces between collagen fibres (Fig. 4E). These voids were subsequently infiltrated by granulation tissue within which numerous small vessels were noted (Fig. 4F). From d21 onwards, there was progressive maturation of granulation tissue becoming fibrotic with reduced vessel density (Fig. 4G). Remaining vessels were disorganised, irregular and small. CG was clearly recognisable in histological sections 4 weeks after injury (Fig. 4H). Seven days after implantation, collagen within autografts appeared to degenerate, the epidermis showed patchy necrosis and blood vessels were collapsed. There was concurrent formation of granulation tissue at the margin between subcutaneous fat and autograft (Fig. 5A). In many areas granulation tissue abutted collagenous autograft ECM, limiting infiltration of host cells. However, where autograft vessels reached the host/graft interface, channels of communication between granulation tissue and perivascular connective tissue were established. This was reflected by progressive perivascular oedema, inflammation and infiltration of granulation tissue within autografts. Graft/host anastomoses subsequently formed, facilitating autograft revascularisation (Fig. 5B and C). Autografts were recognisable histologically 28 days after injury by a lack of fibrosis compared to surrounding tissue (Fig. 5D). DCD samples showed similar behaviour with granulation tissue formation at the margin between subcutaneous fat and DCD in the week after injury (Fig. 5E). Collagen fibres within DCD and adjacent to granulation tissue limited cellular infiltration of the graft. However, decellularised vascular channels reaching the host/graft interface represented an access point for native cells. Consequently, there was rapid influx of endothelial and inflammatory cells through these entry points within 7 days (Fig. 5F). This was followed by incremental infiltration of granulation tissue into these channels (Fig. 5G) similar to that seen in autografts giving rise to a vascular network with red blood cells observed in multiple large vessels after 21 days (Fig. 5H). DCD’s collagen network was progressively colonised by migrating host fibroblasts and myofibroblasts and acted as a skeletal framework for the re-vascularised graft. 4 weeks after injury DCD closely resembled native tissue. Macroscopic changes in wound appearance at each time point can be seen in S1 Fig.

### Whole genome microarrays demonstrate differential up-regulation of angiogenesis-related genes between treatment groups

Whole genome analysis identified 1689 genes with significantly altered expression after injury (S1 Table). After reviewing this initial dataset, 445 of these genes were found to be uniquely up or down-regulated by one treatment modality (control—123, CG— 178, DCD— 144, autograft—0). Gene function for this smaller group was studied and only 4 genes were associated with angiogenesis. They were prokineticin 2 (PROK2), hypoxia-induced translation factor 2A and 3A (HIF2A and HIF3A) and membrane-type 6 matrix metalloproteinase (MT6-MMP) (S2 Table). Recognised genes associated with angiogenesis including angiopoietin 2, hypoxia-induced translation factor 1 and interleukin 1β were excluded because significantly altered expression was observed in more than one treatment group.

### qRT-PCR confirms significant up-regulation of PROK2 and MT6-MMP but not HIF2A or HIF3A mRNA expression in DCD samples compared to controls

**Figure 6:**
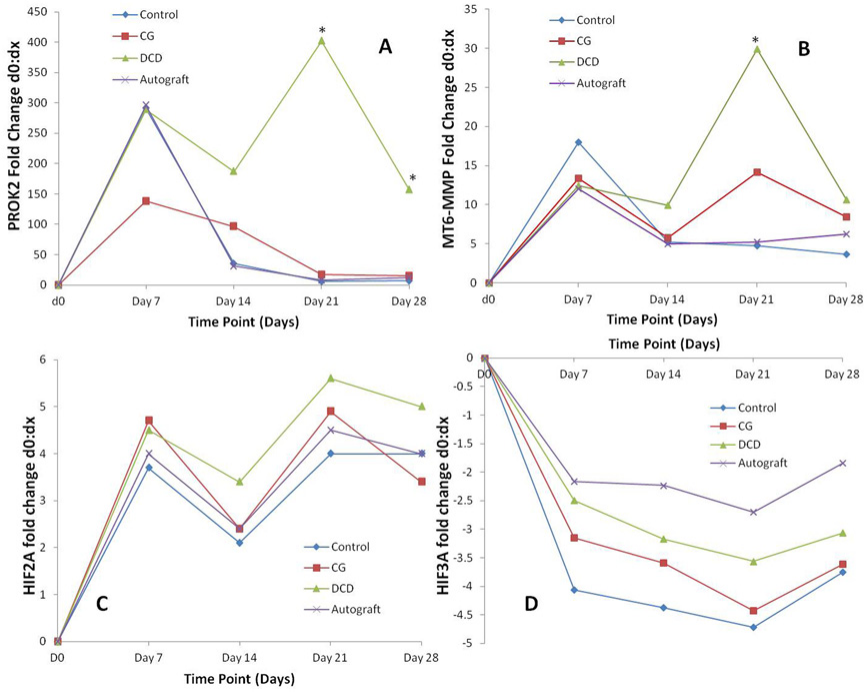
Gene expression profiling. Changes in mRNA expression of prokineticin 2 (PROK2) (n = 5/treatment group) (A), membrane type 6 matrix metalloproteinase (MT6-MMP) (n = 5/treatment group) (B), hypoxia-induced translation factor 2A (HIF2A) (n = 5/treatment group) (C) and hypoxia-induced translation factor 3A (HIF3A) (n = 5/treatment group) (D) after cutaneous wounding derived from qRT-PCR. DCD promotes significant late up-regulation of PROK2 and MT6-MMP. * p<0.05 indicating significant differences between treatment groups at set time points.

There was significantly increased expression of HIF2A, PROK2 and MT6-MMP after injury in all treatment groups (all p = 0.028 at d7). Significantly reduced HIF3A expression was observed at d7 in DCD and control groups (both p = 0.028) as well as d21 in CG samples (p = 0.043). However, HIF3A down-regulation in autografts did not reach statistical significance (p = 0.08–0.14). Furthermore, there was a statistically significant difference in PROK2 expression between treatment groups on d21 and d28 (p = 0.021 & p = 0.004) with DCD values significantly greater than other treatments. On d28 PROK2 expression was also significantly greater in CG and autografts compared to controls (p = 0.043) (Fig. 6A). There was a statistically significant difference in MT6-MMP expression between treatment groups on d21 (p = 0.009) (Fig. 6B). Pairwise comparisons showed DCD values were significantly greater than controls and autografts, whilst CG was significantly increased compared to autografts (all p = 0.043) (Fig. 6B). However, there was no significant difference in HIF2A (p = 0.13–0.71) or HIF3A (p = 0.10–0.75) (Fig. 6C & D respectively) expression between treatment groups.

### Blood vessel number is significantly greater in DCD samples compared to controls using CD31 immunohistochemical staining

**Figure 7:**
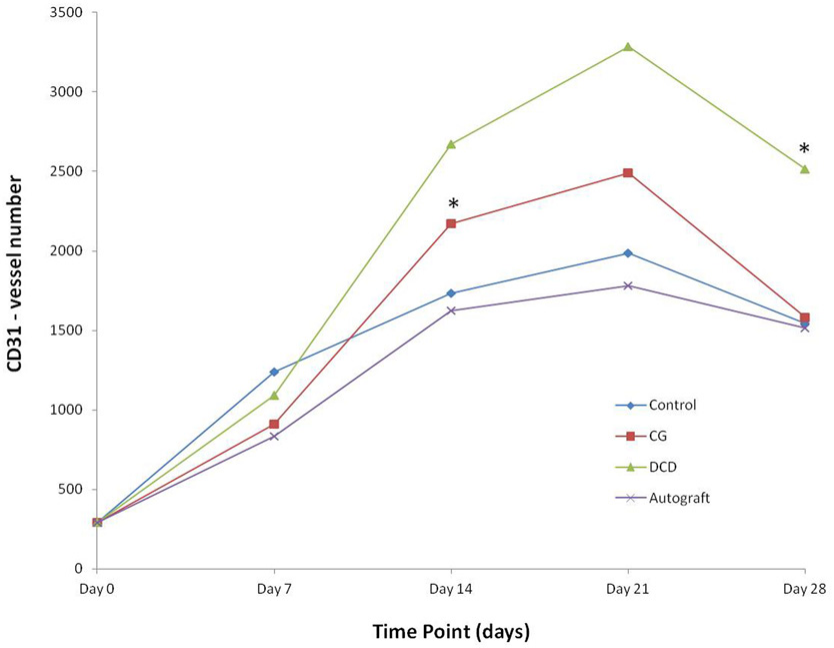
Immunohistochemical staining for endothelial cell marker CD31. Alterations in CD31staining enabling calculation of vessel number after injury which was significantly greater in DCD after 28 days (C) (n = 10/treatment group). * p<0.05.

**Figure 8:**
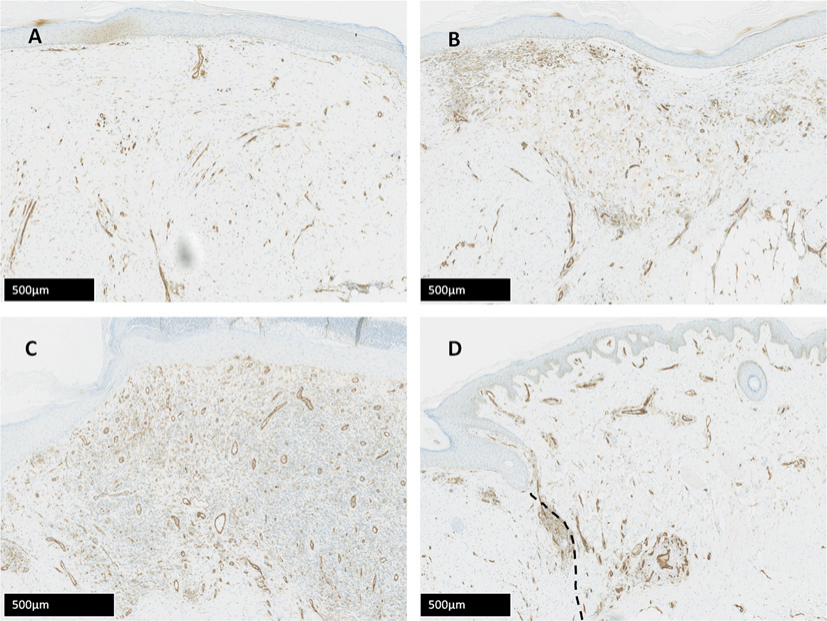
Immunohistochemical evaluation. Visualisation of time-matched (all d28) differences in CD31 expression between treatment groups in the same subject demonstrating significantly greater expression in the DCD treated wound. CD31 stain quantification assessed with Definiens Tissue Studio software and expressed as average vessel number/section. A—Control, B—CG, C—DCD, D—Autograft. Black dotted line—demarcates boundary between autograft and native tissue.

CD31 levels changed significantly after injury in all treatment groups (all p<0.001). Statistically significant differences in vessel number between treatment groups were found on d14 when CG was greater than autografts (p = 0.016), and at d28 when DCD was significantly raised in comparison to controls (Fig. 7 and Fig. 8A-D).

## Discussion

New blood vessel formation contributing to development of granulation tissue is a critical component of wound healing, requiring tightly regulated dynamic reciprocity between endothelial cells, bioactive molecules and ECM components [20]. The structure and composition of ECM alters significantly during wound healing. Consequently its effect on angiogenesis is variable secondary to protein constituents, actions of proteases upon ECM and ECMs ability to sequester cytokines and growth factors [21]. The influence of dSS on this process is still unclear. They significantly change the wound microenvironment by providing a 3-dimensional replacement scaffold material, which may alter expression of growth factors, cytokines and chemokines resulting in different angiogenic behaviour. The majority of data pertaining to dSS use in acute wounds is derived from animal studies, which are hindered by small sample numbers, poor study design and limited translation to human subjects [16, 17, 22]. In contrast this human study is the first to compare multiple treatment types in the same individuals over 6 weeks following cutaneous injury.

DCD, which is derived from human cadaveric split-thickness skin grafts, exerted the strongest pro-angiogenic influence, particularly 3–4 weeks post injury. The same effect was noted previously utilising DCD in treatment-resistant leg ulcers [15]. Collagen density and tensile strength of tissue produced by the decellularisation process is not significantly different from normal skin [19]. Thus, DCD has comparable biomechanical properties to excised native dermis. This is crucial since scaffold characteristics including elasticity, porosity and biocompatibility mediate cellular intrusion, proliferation and differentiation during healing [23, 24]. Furthermore, ECM facilitates transduction of mechanical forces between enclaves of endothelial cells at considerable distance. This establishes tension based guidance pathways enabling formation of large multicellular structures at distance without initially requiring cell to cell contact [25–28]. Thus, it appears the structural similarity between DCD and native dermis enabled rapid progression from isolated groups of endothelial cells scattered throughout DCD on d7 to organised functional vascular networks seen histologically by d21.

By the same logic, autografts should have exerted an equally strong pro-angiogenic effect but we found reduced markers of angiogenesis in this group. This may be because autografts are markedly different to DCD with a full cellular complement, intact dermal vascular architecture and a reservoir of growth factors. Previous studies in mice showed autograft revascularisation is a two stage process. Inosculation between existing graft and wound bed vessels is predominant early on, helping to restore tissue viability. This is associated with a later, limited neovascularisation response producing new vessels between wound bed and autograft [29–31]. Our study made similar findings with restoration of autograft circulation through formation of graft/host anastomoses seen histologically from d7 onwards. This resulted in reduced angiogenic drive evidenced by decreased haemoglobin flux and PROK2 gene expression. However, vessel number was seen to increase up till d21 and this may reflect the outcomes of neovascularisation.

Development of vascular architecture after treatment with autografts and DCD was similar, with both predominantly re-establishing pre-existing vascular channels via defined entry points at the graft-host interface. However, autografts contained functionally intact vessels whilst DCD required complete reconstitution of the vascular network through endothelial cell migration and proliferation. Subsequent differences in angiogenic behaviour may consequently have been due to earlier restoration of blood flow, altered cellular recruitment and secretion profiles as well as reduced neovasculogenesis in autograft samples. By contrast, the looser matrix structure of CG resulted in uniform infiltration of host granulation tissue without any of the prescribed pathways seen in DCD and autografts. Consequently, numerous disorganised granulation tissue vessels developed de-novo, which were rapidly replaced by fibrotic tissue.

The relationship between angiogenesis and fibrosis during wound healing is complex. Evidence is growing that application of angiogenic inhibitors to acute wounds such as VEGF-R2 blockers result in reduced scar formation, whilst pro-angiogenic molecules including IL-8 and TGF-β are found at lower concentrations during scarless foetal healing [32–34]. Autografts in this study provide further supportive evidence for this theory as they were associated with reduced angiogenic drive after graft viability was re-established (between day 0 and day 7) and significantly reduced dermal fibrosis throughout follow-up. It is harder to explain the association between significantly increased angiogenesis and what appeared to be reduced scar formation in DCD samples compared to control and CG modalities. Biomechanical similarities and analogous matrix architecture between DCD and autografts may partly offset pro-fibrotic stimuli. The nature of regenerated dermal vasculature may also be important as control and CG samples both developed large areas of granulation tissue which matured to form dense regions of fibrosis, whilst DCD reconstituted vessel networks cleared during the graft decellularisation process and in turn gave rise to vascular architecture akin to uninjured skin.

Whilst histology demonstrated structural characteristics of dSS influenced subsequent vascular architecture, microarray studies revealed variations in gene expression that may underlie differential angiogenic behaviour. Many pro-angiogenic effects of hypoxia are mediated through hypoxia-inducible transcription factors (HIFs) which control expression of important genes including VEGF, endothelial nitric oxide synthase and heme oxygenase-1 [35]. HIFs are αβ dimers, and the human α subunit has 3 isoforms (1A, 2A and 3A) [36]. HIF1A and HIF2A are well recognised pro-angiogenic molecules though their target genes differ in a temporal manner, HIF2A becoming predominantly expressed during longer periods of hypoxic stress [37]. Indeed, this study showed prolonged up-regulation of HIF2A in all treatment modalities, with greatest expression in DCD samples, though differences between groups did not reach significance. This may be secondary to small sample numbers and natural variation between subjects. HIF3A is less well characterised and its function still unclear though it is not thought to be up-regulated by hypoxia [38–40]. Indeed, most studies show an inhibitory HIF3A effect on HIF dependent gene regulation and consequently an anti-angiogenic action [37]. Our findings support this theory with qRT-PCR data confirming universal HIF3A down-regulation after injury.

PROK2 expression in DCD samples was uniquely up-regulated for a second time 21 days after injury, coinciding with maximal CD31 expression, haemoglobin flux and mean oxyhaemoglobin levels. The 2 forms of prokineticin (PROK1 and 2) are potent angiogenic factors and mitogenic stimulants for endothelial cells that act independently of VEGF [41–43]. They exert their effect through two G-protein coupled receptors (PKR1 and 2) of which PROK2 is the more potent agonist [44]. Guilini et al demonstrated that PROK2 enhanced endothelial cell proliferation and migration using wound healing assays. Furthermore PROK2 action upon PKR1 promoted vessel-like formation via PI3K/Akt and MAPK signalling pathways [41]. PCR concurrently demonstrated a similar bimodal pattern of mRNA expression for MT6-MMP in DCD samples. This multifunctional enzyme is capable of cleaving numerous ECM proteins and there is growing evidence that MT-MMPs (particularly MT1-MMP) are critical regulators of endothelial cell invasion into 3-dimensional collagen or fibrin matrices, and promote endothelial cell tubular morphogenesis [26, 45, 46]. Thus we suggest there is likely to be an innate response to injury, irrespective of treatment modality reflected by formation of granulation tissue with early (d0–7) up-regulation of genes such as HIF2A, PROK2 and MT6-MMP in all treatment groups. However, progressive ordered colonisation of DCD by host cells generates a second response from day 14 facilitating endothelial cell proliferation and migration resulting in increased vessel formation in only these samples.

In conclusion, this study compared angiogenesis in control wounds with those treated using CG, DCD and autograft in healthy individuals. Angiogenic responses and neovascular architecture post-wounding were variable between treatment groups suggesting behaviour is strongly influenced by composition and biomechanical characteristics of matrix materials. There is clear evidence from invasive and non-invasive modalities that treatment with DCD resulted in increased angiogenesis. Significantly elevated mRNA expression of pro-angiogenic PROK2 and ECM protease MT6-MMP seen only in the DCD group may contribute to the observed response.

## Supporting Information

S1 Table Spreadsheet of 1689 genes identified with significantly altered expression after injury derived from Qlucore whole genome microarray analysis. (XLS)

S2 Table Characteristics of genes related to angiogenesis identified by microarray studies. (DOCX)

S1 Fig Characteristic changes in macroscopic appearance of wounds in each treatment group at successive time points. All images in this figure are derived from the same patient. CG—collagen-GAG scaffold, DCD—decellularised dermis. Scale bar is identical in each image.
(TIF)
