# Correction: Acute Cutaneous Wounds Treated with Human Decellularised Dermis Show Enhanced Angiogenesis during Healing

**DOI:** 10.1371/journal.pone.0121503

**Published:** 2015-03-23

**Authors:** The PLOS ONE Staff

Dr. Syed A. Iqbal should be included in the author byline. He should be listed as the second author, and his affiliation is 1: Plastic and Reconstructive Surgery Research, Manchester Institute of Biotechnology (MIB), The University of Manchester, Manchester, Lancashire, UK. The contributions of this author are as follows: Performed the experiments, analyzed the data. The correct citation is: Greaves NS, Iqbal SA, Morris J, Benatar B, Alonso-Rasgado T, Baguneid M, et al. (2015) Acute Cutaneous Wounds Treated with Human Decellularised Dermis Show Enhanced Angiogenesis during Healing. PLoS ONE 10(1): e0113209. doi:10.1371/journal.pone.0113209

The first author, Dr. Nicholas S. Greaves, should not be listed as a Corresponding Author. The sole Corresponding Author should be Dr. Ardeshir Bayat.
