# Supplementary material for: Acute Cutaneous Wounds Treated with Human Decellularised Dermis Show Enhanced Angiogenesis during Healing
Source: PLoS One. 2015 Jan 20;10(1):e0113209. doi: 10.1371/journal.pone.0113209 (PMC4300088; doi:10.1371/journal.pone.0113209)
Supplement: S2 Table — (DOCX) [file pone.0113209.s002.docx]

| **Gene Symbol** | **Forward Primer** | **Backward Primer** | **Probe number** | **p-value** | **q-value** | **Fold change**  **d0vsdx** | **Gene name** |
| --- | --- | --- | --- | --- | --- | --- | --- |
| **HIF2A/EPAS1** | cggagtctagcgcatggta | gacatgaagttcacctactgtgatg | 17 | 1.58E-05 | 0.0024 | 0.420802 | Hypoxia-Inducible factor 2, alpha subunit/ Endothelial PAS domain protein 1 |
| **HIF3A** | gctcattcaggttcaggagtg | ctttctgctctttcctctcagc | 45 | 0.0012 | 0.9999 | 2.11098 | Hypoxia-Inducible factor 3, alpha subunit |
| **MT6-MMP/ MMP25** | tggccaaactcatggacag | ctccggggacactcacttt | 85 | 7.96E-06 | 0.0017 | 0.231164 | Membrane-type 6 matrix metalloproteinase / Matrix Metallopeptidase 25 |
| **PROK2** | cagacatgggcaagtgtga | tgccatccactgactcgtaa | 88 | 1.02E-05 | 0.0018 | 0.039470 | Prokineticin 2 |

Supporting Information Table S2. Characteristics of genes related to angiogenesis identified by microarray studies.
